# An imported malaria case with repeated episodes of neurological syndromes resulting from different *Plasmodium* species

**DOI:** 10.1186/s12879-023-08872-y

**Published:** 2024-01-03

**Authors:** Zheng Xiang, Longcan Zhou, Maohua Pan, Yucheng Qin, Yao Bai, Pien Qin, Weilin Zeng, Xiaosheng Wei, Yuxin Lu, Charurut Somboonwit, Lynette Menezes, Yaming Huang, Liwang Cui, Zhaoqing Yang

**Affiliations:** 1 Department of Pathogen Biology and Immunology, Kunming Medical University, Kunming, Yunnan Province China; 2 Shanglin County People’s Hospital, Shanglin, Guangxi China; 3 Department of Internal Medicine, Morsani College of Medicine, University of South Florida, 3720 Spectrum Boulevard, Suite 304, Tampa, FL 33612 USA; 4 Guangxi Zhuang Autonomous Region Center for Disease Control and Prevention, Nanning, Guangxi China

**Keywords:** Cerebral malaria, *Plasmodium falciparum*, *Plasmodium vivax*, Post-malaria neurological syndrome, Sequential episodes, West Africa, Submicroscopic infection, Artesunate treatment

## Abstract

**Background:**

Imported cerebral malaria (CM) cases in non-endemic areas are often misdiagnosed, which delays treatment. Post-malaria neurological syndrome (PMNS) after recovery from severe malaria can also complicate diagnosis.

**Case:**

We report an imported malaria case from West Africa with two sequential episodes with neurological syndromes within about a month. The first episode was diagnosed as CM with microscopy-positive *Plasmodium falciparum* infection. The second episode, occurring a month after the recovery from the first CM episode, was consistent with PMNS, since malaria parasites were not detected by microscopy in peripheral blood smears. However, this diagnosis was complicated by the detection of *Plasmodium vivax* in peripheral blood by PCR, suggesting a potential cause of the second episode by *P. vivax.*

**Conclusion:**

This study suggests that PMNS often occurs after severe falciparum malaria. Concurrent *P. vivax* infection with pathogenic biomass being predominantly extravascular further complicates accurate diagnosis.

**Supplementary Information:**

The online version contains supplementary material available at 10.1186/s12879-023-08872-y.

## Background

Cerebral malaria (CM) is the most common and serious neurological complication arising mostly from infection with *P. falciparum* [1, 2]. Mounting evidence also confirms *P. vivax* as another major cause of severe malaria despite relatively low-grade parasitemia in peripheral blood [3, 4]. The most common symptoms of severe vivax malaria include severe anemia, acute respiratory distress syndrome, impaired consciousness, and renal failure [5, 6]. Severe vivax malaria has been reported disproportionally from endemic countries [5]. Reviews of inpatient records in hospitals of western Cambodia and the Thailand-Myanmar border showed that severe vivax malaria, though relatively rare, was also an important cause of hospitalization [7, 8]. Likewise, vivax CM cases, although not frequently documented, have been reported mostly from India and Pakistan [5, 6, 9, 10]. Despite proper antimalarial treatment, CM still carries a 15–20% mortality rate in children and 30% in adults [11].

Since the symptoms of CM can deteriorate rapidly, timely diagnosis and effective treatment are vital for rescuing CM patients. Unfortunately, imported CM cases in non-endemic areas are often misdiagnosed, and delayed treatment leads to adverse and fatal outcomes. Here, we report an unusual malaria case in southern China imported from West Africa, where the patient experienced two sequential cerebral episodes within one month, which may be caused by microscopically patent *P. falciparum* and tenebrous *P. vivax*, respectively.

## Case history

**Fig. 1:**
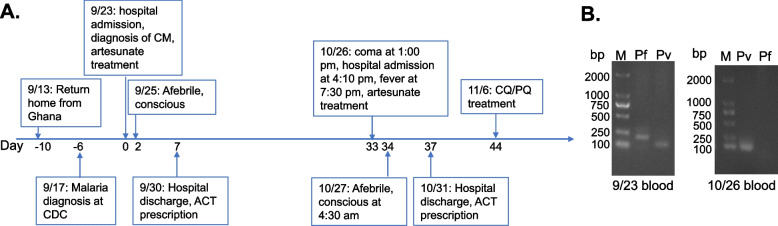
**A** Chronological events of the malaria case. The day of his first hospital admission was designated as Day 0. **B** Gel images of the PCR diagnosis of the patient’s two blood samples collected during his two hospital admissions targeting the *18S rRNA* gene. M – molecular marker in bp; Pf – *P. falciparum* (205 bp); Pv – *P. vivax* (120 bp)

A 34-year-old male attended the general outpatient clinic of Shanglin County Hospital, Guangxi, China, on September 23, 2016 (Fig. 1A). The night before, the patient developed systemic pain and discomfort in the epigastric area. As his symptoms intensified by the morning, he sought medical attention at the hospital around noon. Because of his primary complaint of epigastric pain and no obvious abnormalities from the physical examination, an esophagogastroscopy was performed, but it did not identify any significant pathology except hyperemia and edema of the gastric mucosa. One hour post gastroscopy, the patient suddenly became confused and disoriented and was immediately referred to the emergency unit. Physical and laboratory examination revealed that the patient weighed 50 kg with a BMI of 18.4 kg/m^2^, slightly underweight. He was febrile (39.5℃), tachycardic (pulse rate of 111 times/min), and thrombocytopenic (Table 1).

**Table 1:** Laboratory test results

**Tests**	**1**^**st**^** admission**^a^				**14 days**	**2**^**nd**^** admission**	
	**0 h**	**12 h**	**44 h**	**96 h**		**0 h**	**6 h**
Axillary temperature (°C)	39.5	-	37.5	36.5	36.5	38.5	38.0
**Blood tests** (reference range)							
White blood cells (10^3^/µL) (4.5–11.0)	3.88	8.32	10.54	7.41	7.59	8.28	8.34
Red blood cells (10^6^/µL) (4.09–5.74)	4.71	4.31	3.66	4.01	4.29	4.27	4.18
Hematocrit (%) (38–50.8%)	39.6	35.7	30.6	35.0	37.7	38.7	37.7
Hemoglobin (g/dL) (13.1–17.2)	13.8	12.5	10.5	11.4	12.3	12.5	12.5
Platelet (10^3^/μL) (130–400)	32	27	97	210	498	255	252
Lactate dehydrogenase (U/L) (140–280)	-	1,096	-	800	-	180	-
Total bilirubin (µMol/L) (0–17)	-	23	-	5.6	5.9	8.0	-
Parasite detection ^b^	++++	+	-	-	-	neg	-
Nested PCR	Pf/Pv	-	-	-	-	Pv	-
**Urinalysis** (reference range/µL)	**0 h**	**7 h**	**15 h**	**88 h**		**0 h**	**6 h**
White blood cells (0–20)	31	21	63	3.4	-	-	-
Unclassified casts (0–1)	1.9	0	0	0	-	-	-
Urobilinogen (–)	1+	neg	neg	neg	-	-	-
Bilirubin (–)	1+	neg	neg	neg	-	-	-
Urine occult blood (–)	3+	3+	3+	neg	-	-	-
Protein (–)	2+	2+	2+	2+	-	-	-
Nitrite (–)	+	+	+	neg	-	-	-

^a^Time after the malaria diagnosis

^b^Parasite density per µL blood detected by microscopy (time after treatment): 3.2 x 10^4^ (0 h), 2.4 x 10^4^ (4 h), 1.6 x 10^4^ (8 h), 8 x 10^3^ (12 h), 6 x 10^3^ (24 h), 5 x 10^3^ (28 h), 10 (36 h), 0 (44, 52, 56, and 89 h)

-, Not done; Pf, *P. falciparum*; Pv, *P. vivax*

An accompanying family member provided medical information and travel history. The patient had worked in Ghana since August 2014 and returned to his home in China ten days before the presentation. During his stay in Ghana, he did not take chemoprophylaxis, had suffered from malaria several times, and had been treated locally. The routine malaria screen by microscopy at the County CDC 7 days before his presentation (September 17, 2016) was *Plasmodium*-negative. Given his travel history and symptoms, he was suspected of having a malaria infection. Microscopy of his blood smear confirmed *P. falciparum* infection with a parasite density of 3.2×10^4^/μl. With the diagnosis of CM, rescue treatment with intravenous artesunate (loading dose of 120 mg, and subsequently 60 mg/4 h) was immediately initiated. Five hours after admission, he was in a coma and unresponsive, with a Glasgow coma score of 4 (No eye opening, +1; no verbal response, +1; extend motor response, +2). He exhibited shallow breathing and had coffee-colored urine. Blood LDH level and urinalysis also indicated massive hemolysis (Table 1). He was transferred to the intensive care unit and rapidly required intubation for mechanical ventilation. At 12 h, he remained thrombocytopenic with elevated lactic acid dehydrogenase and bilirubin (Table 1). Parasitological monitoring every 4-8 h showed that his parasitemia continually decreased during the treatment. At 44 h, the patient became aparasitemic, slightly febrile (37.5°C), and more alert. The mechanical ventilation was weaned, and artesunate injection was adjusted to 60 mg/12 h. At 66 h, he returned to his baseline mental status and was transferred to the inpatient unit, where his condition continued improving. Seven days after admission, he was discharged and prescribed a full course of dihydroartemisinin-piperaquine oral tablets for the subsequent three days. Seven days later, a blood examination showed that he was parasite-negative and had made a good recovery (Table 1).

At 1:00 pm on October 26, 2016, he collapsed while working at home. He developed generalized seizures and became unresponsive (Fig. 1A). He was rushed to the county hospital. Physical, routine laboratory and head CT examinations revealed no obvious abnormalities (Table 1). He was unconscious with a Glasgow coma score of 4, lethargic, and irritable. Microscopy of peripheral blood thick smear (400 ×, 100 fields) showed no *Plasmodium*. The patient developed a fever (38.5℃) at 7:30 pm, following which he was presumptively administered intravenous artesunate because of possible malaria recrudescence. The next morning (4:30), the patient became afebrile and regained consciousness. Two days after admission, the patient's vital signs returned normal. Antimalarial therapy was discontinued, and blood smears examined every 8 h were *Plasmodium*-negative. Five days after admission, the patient was discharged with a course of artesunate-amodiaquine oral tablets.

The patient's blood samples at the two admissions were sent to Kunming Medical University for molecular diagnosis. Nested PCR [12] detected mixed *P. falciparum/P. vivax* infections during his first episode and *P. vivax* for the second (Fig. 1B). With a normal glucose-6-phosphate dehydrogenase level, he received a course of oral treatment with chloroquine (1500 mg/3 days) and primaquine (22.5 mg/day for eight days) beginning 11 days after his second episode to prevent future relapse. He remained healthy throughout a two-year follow-up.

## Discussion

Prompt diagnosis and treatment of malaria are challenging in non-endemic countries, and delays are associated with increased risks of severe disease and mortality [13]. In our case, travel history played a critical role in the diagnosis. Since malaria was eliminated in Guangxi in 2012 [14] and the patient presented with atypical malaria symptoms at his first admission, diagnosis for malaria was delayed until his symptoms rapidly deteriorated to a comatose condition. It was his African travel history that alerted the doctors and led to the diagnosis of *P. falciparum* CM. Since 2012, treating thousands of returning expatriates with imported malaria infection from Africa provided adequate training to the medical doctors in the Shanglin County Hospital. Increased awareness and preparedness of doctors regarding early detection and treatment of imported malaria in non-endemic countries are desired.

Compared to the WHO definition of CM, which requires the identification of asexual *Plasmodium* parasitemia in peripheral blood, the second episode of neurological syndrome resembles the post-malaria neurological syndrome (PMNS), a complication occurring within two months after recovery from a severe *P. falciparum* attack without detecting malaria parasites in the blood [15–17]. PMNS is a predominantly self-limiting condition with a median duration of symptoms of 13 days (range 3-25 days) [16]. However, this diagnosis was complicated by the detection of *P. vivax* by PCR, which suggested that the second episode might be due to *P. vivax* infection. It could be argued that the PCR result for the second episode might be from the persisting parasite DNA from the first episode of active infection, but the PCR detection of *P. vivax* but not *P. falciparum* (the predominant infection during the first episode) argues in favor of active malaria. Besides, the patient responded rapidly to artesunate without antibiotic treatment (compared to the relatively slower process of PMNS), suggesting *Plasmodium* and excluding bacterial meningitis as a probable cause of the second cerebral episode. Thus, despite the patient only had submicroscopic *P. vivax* parasitemia in peripheral blood, the fact that *P. vivax* may have pathogenic biomass that is predominantly extravascular may explain his second episode, which is consistent with the definition of severe tenebrous vivax malaria with cerebral syndrome [18].

This case demonstrates the risk of both *P. falciparum* and *P. vivax* as the cause of severe malaria in travelers to Africa. Since coma associated with *P. vivax* mono-infection is much less frequent than falciparum malaria [19], the finding of imported *P. vivax* infection from West Africa as the potential underlying cause of his second episode is unexpected but consistent with the increasing detection of vivax malaria in Duffy^–^ populations. Whereas *P. vivax* was not detected in surveys in Ghana [20], reports of *P. vivax* infections in travelers/migrants from Ghana suggest the existence of *P. vivax* transmission there [14, 21]. In addition, with the hidden reservoir of *P. vivax* in extravascular space, a combination of microscopy with serological and molecular tests is needed to diagnose *P. vivax* infections in returning travelers. As imported relapsing malaria incidence continues to rise in non-endemic regions [14], this case illustrates the need for heightened awareness among healthcare providers to consider *P. vivax* alone or as a mixed infection with *P. falciparum* in the absence of detectable vivax parasitemia as the potential cause in patients with a travel history and altered mental status.

This case report has revealed several limitations with the current malaria diagnosis and treatment policy in China. The lack of a comprehensive febrile diagnostic work-up and reliance on microscopy for malaria diagnosis will certainly overlook non-malarial infections and submicroscopic malaria, as presented in this case. Notably, the severe malaria case was treated with artesunate infusion with a shortened interval. Despite promising results from treating many severe malaria cases in Shanglin Hospital, the regimen requires vigorous clinical studies. In addition, uncomplicated falciparum malaria in China is treated with an artemisinin-based combination therapy without a single low-dose primaquine. Although this may appear justified for imported malaria in areas without vectors, adding the low-dose primaquine should be advocated, especially in southern China, where malaria has recently been eliminated, and vectors are abundantly present.

### Supplementary Information

**Additional file 1: Sup Fig. 1.** Agarose gel images of the PCR diagnosis of the patient’s blood samples collected during his two hospital admissions targeting the 18S rRNA gene. M – molecular marker in bp; Pf – P. falciparum (205 bp); Pv – P. vivax (120 bp).

## Data Availability

The datasets used and analysed during the current study are available from the corresponding authors upon reasonable request.
