# Supplementary material for: An imported malaria case with repeated episodes of neurological syndromes resulting from different Plasmodium species
Source: BMC Infect Dis. 2024 Jan 3;24:41. doi: 10.1186/s12879-023-08872-y (PMC10763073; doi:10.1186/s12879-023-08872-y)
Supplement: Supplementary file 1 — Additional file 1: Sup Fig. 1. Agarose gel images of the PCR diagnosis of the patient’s blood samples collected during his two hospital admissions targeting the 18S rRNA gene. M – molecular marker in bp; Pf – P. falciparum (205 bp); Pv – P. vivax (120 bp). [file 12879_2023_8872_MOESM1_ESM.docx]

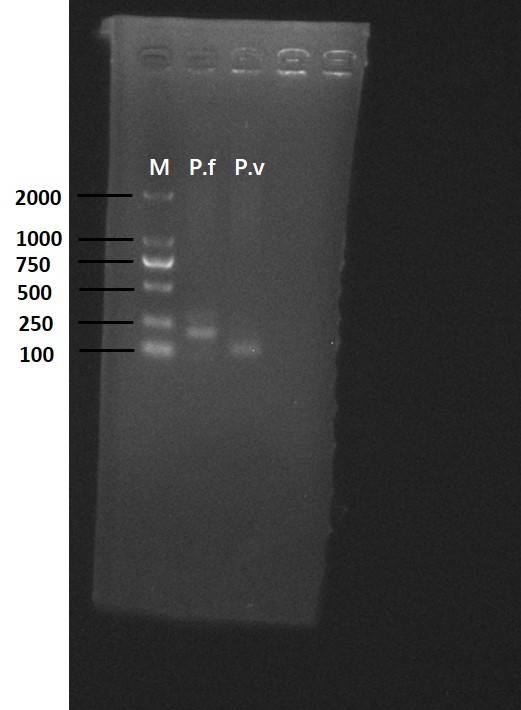

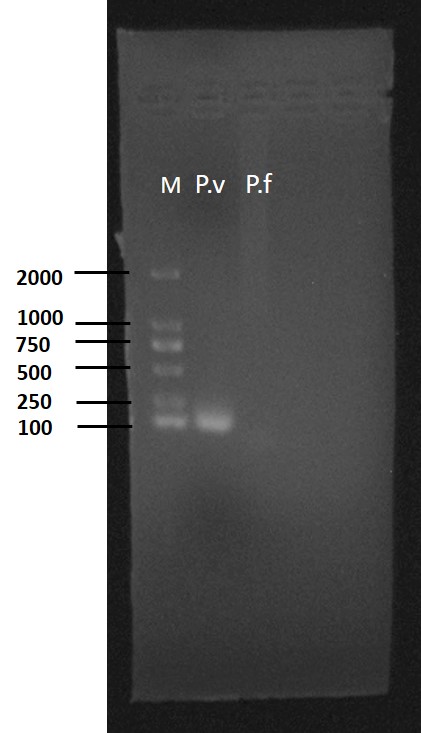


Sup Fig.1 Agarose gel images of the PCR diagnosis of the patient’s blood samples collected during his two hospital admissions targeting the 18S rRNA gene. M – molecular marker in bp; Pf – P. falciparum (205 bp); Pv – P. vivax (120 bp)
